# Round the Hospitals

**Published:** 1918-08-31

**Authors:** 


### ROUND THE HOSPITALS.

As we go to press the news reaches us that London is shortly to enter into possession of a Local Centre of the College of Nursing. The activities of College members all over the kingdom have fired many in London with the desire to possess their own club-house and a meeting-ground for debate, discussion, and professional lectures. It is quite certain that one Centre will not satisfy the aspira-

**ROUND THE HOSPITALS—(continued).**

tions of London members. Four, at the least, or even five would seem to be required if the club-house is to be more than an arduous luxury attained to occasionally after tedious journeyings. But everything must have a beginning, and the projected London Centre may be regarded as a forerunner. London can never be regarded from the same standpoint as that of even the most considerable cities in the provinces. A central club-house with accommodation not merely for dwellers near by, but for all College members on their way through the Metropolis, and with spacious lecture-halls and assembly-rooms, is part of the original plan of the College, and other London Centres, once this is in being, will follow on in due course.

The position at the moment is that the College of Nursing has long outgrown the temporary premises in Vere Street, and that the imperative need for more space is making itself felt simultaneously with the impulse to provide London members with a meeting-ground. The Council have had the double problem before them. They have inspected various available premises and made considerable progress in the critical business of selection. All that we are allowed to say at present is that the quest has reached a very hopeful stage. There is every prospect that before the winter work begins a building may be secured ample for the accommodation of the business department of the College, as well as for the projected London club-house. The desirability of having all under one roof is obvious. A special meeting of the College of Nursing will be held in London early in September to consider the arrangements to be made for the London Centre. We hope to announce the date of this meeting in a subsequent issue.

The Honours in connection with the military operations in Mesopotamia have been issued this week (dated June 3, 1918). They include the award of the Royal Red Cross to the following in recognition of their valuable services with the British Forces in Mesopotamia:—First Class.—*Queen Alexandra's Imperial Military Nursing Service Reserve*: Sisters M. M. McNab, A.R.R.C., M. Rae (Acting Matron), E. S. Wilkinson. *Territorial Force Nursing Service*: Matron A. L. Earle; Sisters M. G. Coulson, M. K. Wheeler. *Queen Alexandra's Military Nursing Service for India*: Matron M. G. Gilmore. Second Class.—*Queen Alexandra's Imperial Military Nursing Service Reserve*: Sisters C. M. Bottomley, A. L. Hartick, M. A. Robertson, S. E. Wadsworth; Staff Nurses M. B. Argo, A. M. Davies, E. Davies, A. E. Reid, A. Wellington. *Territorial Force Nursing Service*: Sisters M. F. D. Crosbie, N. Curties, E. A. Emuss, M. Hunstone, B. E. Seacome; Staff Nurse E. S. King. *Queen Alexandra's Military Nursing Service for India*: Nursing Sisters M. E. Maclean, E. O. Marshall. General Marshall's dispatch of April 15, 1918, published as we go to press, includes the names of several ladies whose distinguished and gallant service and devotion to duty he considers deserving of special mention. We regret we are compelled, owing to pressure on our space, to hold over the list of names this week.

Mrs. Elizabeth R. Creagh, R.R.C., Matron-in Chief of the South African Military Nursing Service, has been appointed by the King to be an Officer of the Order of the British Empire (O.B.E.), for valuable services rendered in connection with military operations in German South-West Africa, whilst the following have been awarded the Royal Red Cross in recognition of their valuable services in the German South-West African Campaign, 1914-15:—*First Class*: Matron I. G. Alexandra; Nursing Sisters H. H. Weise, A.R.R.C., and E. S. Wessels, A.R.R.C.; Staff Nurses H. L. Bester, A.R.R.C., M. A. Fynn, A.R.R.C. *Second Class*: Nursing Sisters E. Burgess, J. C. Childe, J. N. Ferguson, C. J. Hawkes, A. M. Newth, E. M. Pearson, B. J. Wilde, E. Wilson; Staff Nurses G. Krohn, Mrs. J. Langman (*née* Patterson), D. N. K. Van Niekerk, all of the South African Military Nursing Service. The Governor-General and Commander-in-Chief, Union of South Africa, has brought to the notice of the Secretary of State for the Colonies, for distinguished service in connection with the same campaign, the names of Matron Niven, R.R.C., Nursing Sisters D. Sartis, S. Van der Riet, and G. E. L. Waters; Staff-Nurse B. M. Morris—all belonging to the South African Nursing Service.

The Military Medal has been awarded to Staff-Nurse P. E. Corkhill, Australian Army Nursing Service, for distinguished service in the field and courage and devotion on the occasion of an enemy air-raid. She continued to attend to the wounded while the raid was in progress, and by her example did much to allay the patients' alarm.

M. Pichon, in his address to the Allied Women Workers assembled in Congress at Paris, had much to say on woman's new rôle in the years to come. He declared that woman will no longer be content to leave to man affairs which concern her as much as him. "She will have had such a part in the liberation of the world that the world will not be able to keep her separated from the defence of great social causes." This was in accord with Mr. Lloyd George's declaration that "to woman's ennobling influence we look not only for strength to win the war, but for inspiration during the great work of reconstruction which we shall have to undertake after victory is won." The pessimists who make it their principal occupation to prophesy dark things about the future prospects of women war workers would do well to lay these utterances to heart. The personal service which lies ahead of us in the days of peace is more intricate, more arduous, more stupendous in amount than any which the war has yet demanded.

**ROUND THE HOSPITALS**—*(continued)*.

It appears from the statement of the Deputy Registration Officer at Enfield, in answer to an applicant, that the matron alone, if at all, will be eligible to register as a voter from among the nursing staff in most institutions. The condition for registration is ability to prove that the nurse as part of her contract or engagement is definitely entitled to the use of a special room, and cannot be removed without due notice. This is true pretty exclusively of the matron's quarters. In certain large institutions, however, the assistant matrons and other higher nursing officials have permanent quarters assigned to them, and they should, if entitled to do so, claim their privilege of being inscribed on the register. The difficulty lies in the required stipulation in their contract that " they shall not be removed without due notice "—a most uncommon clause in hospital agreements, but one likely to make its appearance in the future should the vote be keenly desired.

---

The Governors of St. Thomas's Hospital express themselves in the recently issued annual report as " particularly gratified by the honour conferred on our matron, Miss Lloyd Still, who was in the first list of the Order of the British Empire, and was awarded the O.B.E. in recognition of her valuable work for the nursing profession." In addition the following honours have already been awarded to St. Thomas's nurses: Military Medals 3; R.R.C., First Class, 20; R.R.C., Second Class, 24; Foreign Order (Serbia), 1. The Governors put it on record that " all the nursing work has been carried out efficiently in spite of the very many calls made for service abroad, calls which entail frequent changes in the *personnel* and throw even heavier responsibilities than usual upon the matron and her staff. From this hospital no less than 178 nurses are serving in His Majesty's Forces."

---

In striking contrast to the cordial interest and pride in the high level of the Nursing Service manifested by the Governors of St. Thomas's Hospital we have a report put forth by the Board of Management of St. Mary's Hospital, Paddington, in which no single reference to the nursing staff is suffered to appear. Except for an item in the accounts of £3,139 for nursing salaries, and the appearance of the matron's name, Miss H. F. Muriel Jackson, in the list of officers, the nursing staff is absolutely ignored. The chaplain, the almoner, and the Ladies' Association are accorded sympathetic notices. The list of Honours earned by past or present students of the medical school is duly recorded. Have no St. Mary's sisters or nurses, it must be asked, obtained distinctions also? The hospital does less than justice to its fine training-school.

---

Some Dutch sisters, who have been lately in London, have frequently had both German and British wounded under their care on the Dutch hospital ship *Sindoro*, employed on repatriation duty, and have been contrasting the two sets of men, very much to the advantage of our own. They bore sad testimony to the neglect by German nurses of the British under their care, who were frequently found in a very neglected state, " the dirt thick on their bodies." They found the Germans quarrelsome and abusive about their captivity, while the English made no complaints unless interrogated, when they confessed their treatment had been very bad. The Dutch sisters were particularly struck by the pleasant feeling of comradeship between British officers and men on board—a striking contrast to the severe and harsh military system of the Germans, maintained even among the invalids and badly wounded.

---

The grave deficiency which exists at the present moment for in-patient treatment of infants and children has been brought forcibly before the public by the Mansion House Council on Health and Housing, of which the Lord Mayor is president. It has been proposed that all hospitals should either enlarge their children's wards or provide new ones; and that accommodation should be provided in connection with Infant Welfare Centres for receiving cases which require residential treatment. At the present moment many Poor-Law infirmaries are crowded out with children whom they are compelled to house most unsuitably in adult wards. A large proportion of these are from well-to-do homes, and if other accommodation were available payment would be readily forthcoming. Young children respond with lightning rapidity to the change of their environment to a quiet, airy, and hopeful atmosphere after the emotional excitement, over-feeding, and stuffiness too often attendant upon their ailments at home. It seems, indeed, sometimes sufficient to let them lie quiet and recover, and facilities should be provided for this purpose apart from associations with acute forms of illness.

---

Meantime the Lord Mayor's Council reports very unfavourably on the local Poor-Law infirmary facilities for receiving children. They declare that cases are discharged too soon; that babies in particular often come out worse than they went in; that there is a lack of care and cleanliness; a shortage of staff and deficiency of accommodation; indifferent nursing and medical treatment. It is too true that babies have to lie alone for hours in their cots with feeding-bottles fastened to the cribs within reach. If the public would only visit the infirmaries themselves, and ask to see the children's wards, they could get these things altered within six months. It is largely a question of money, money, money.

---

A correspondent in the Scotch press draws attention to the condition attached to the enlistment of women in the Voluntary Aid Detachments of Scotland. For eighteen months, he says, a form has been used under which " these ladies have been

**ROUND THE HOSPITALS—**(*continued*).

asked to sign a declaration by which they bind themselves to the liability of instant dismissal by their Commandant, subject to the approval of their respective County Directors, and that 'without reason assigned.' The specimen I have seen [writes Mr. G. W. Wilton] is countersigned by the chairman and the secretary." After pointing out that no private soldier can be dismissed "without reason assigned," and consequently without a chance to defend himself, the writer says that "objection was taken to the adoption of this peculiar form of declaration," but that it was overruled by a group of martinets. He adds that "no such odious clause is found in the engagements made by the Scottish Branch with the members of their nursing staff," and it is to be hoped that the matter will be sifted and the objectionable clause deleted. In other respects the Scottish regulations bear unfairly on general service members, and it is clear they want thoroughly overhauling.

---

Aldershot Hospital has recently "come of age," and Miss Gregory, who has been matron during the whole period since it was founded, has been presented by the committee of management with a Georgian silver tea service in honour of the occasion, with a salver bearing the following inscription: "Presented to Miss Gregory with the sincere wishes of the President and members of committee, as some mark of their esteem and appreciation of twenty-one years' devoted service as matron of the Aldershot Hospital, 13 August, 1897, 13 August, 1918." The institution, which has seventeen beds, receives probationers for a year's preliminary work, but, of course, issues no certificate. Under a linking system these small hospitals should continue to fill a gap by helping women to find their vocation, and the probationers they prepare for training are likely to be readily absorbed by the training-schools.

---

Dr. Brock, writing in the *Sociological Review* on the restoration of the neurasthenic, has some searching observations on shell-shock hospitals as presenting a microcosm of the modern world. In his view war psychoses are but "an active exacerbation of a more or less chronic sub-acute condition," from which society had been suffering long before the war. For instance, he classes under the head of drug-addiction such habits as over-stimulation of the heat sense by hot drinks, hot baths, extra clothing, over-heated rooms, even hot bottles to the feet at night. Again we have "grousing" or grumbling classed as a form of self-drugging. It is "protective neurosis," for criticising other people tends to raise one relatively in one's own self-esteem, and it is a constant symptom in the shell-shock sufferer. A habit allied to that of grumbling and the curse of institution life, so Dr. Brock declares, is that of standing on one's dignity, properly speaking, another form of drug-taking, for those who lack self-respect instinctively attempt to bolster themselves up by supports which do not call for effort on their part. Now that their true inwardness has been explained it is certain that grumbling, standing on dignity, sitting over the fire, craving for hot tea, and cherishing the nightly hot bottle will speedily cease to be features of institution life.

---

Glasgow is making good progress with its new Nurses' Club, which has enlisted influential support. The appeal for funds lately put forth by the Lord Provost brought in £5,025 at once, about half of the capital sum required in order to place the scheme on a sure foundation, and already good central premises have been secured in Bath Street. The Most Honble. the Marchioness of Ailsa is President; Mrs. J. V. Stewart, Vice-President; Mrs. David McCavan, Convener; Mrs. J. F. Pollock, Vice-Convener; Sir John S. Samuel, Hon. Secretary and Treasurer. When in working order the club is to be managed by a committee of nurses, with whom five representatives of the trustees will be associated. It is not to be identified with any particular organisation for nurses, but is intended for the use of all nurses, whatever association they may belong to, presumably with some stipulations as regards training. The rate of subscription has not yet been fixed.

---

Among the protective measures devised for the better safety of sisters in the war area is the use of khaki-coloured caps and handkerchiefs for such as are on night duty. Nothing more striking and conspicuous can be imagined than the snow-white circle presented to the airman by the nurse's head as she moves swiftly across the quadrangle. And, alas! such an indication of hospital activities below is no longer regarded with reverence by the foe. It merely suggests to him the chance of obliterating a handful of wounded men.

---

The new American Army School of Nursing established by the Surgeon-General, Washington, D.C., offers a course leading to a diploma in nursing for women desiring to care for the sick and wounded soldiers. The military hospitals will provide experience in surgical nursing, including orthopædic, eye, ear, nose, and throat; medical, including communicable, nervous, and mental diseases. Experience in the diseases of children, gynæcology, obstetrics, and public health nursing will be provided through affiliations in the second or third year of the course, which will extend over three years. Credit of nine months or of three or four months respectively will be given to graduates of accredited colleges and technical schools. Those who acquit themselves well and pass the final and other examinations will be eligible for registration in any State if they have completed a full three years in hospital. Already 550 students are taking the summer nursing course at Vassar College with a view to passing into the Army School of Nursing.

